# Perceptions of Heart-Healthy Behaviors among African American Adults: A Mixed Methods Study

**DOI:** 10.3390/ijerph15112433

**Published:** 2018-11-01

**Authors:** Cheryl Der Ananian, Donna M. Winham, Sharon V. Thompson, Megan E. Tisue

**Affiliations:** 1 College of Health Solutions, Arizona State University, Phoenix, AZ 85004, USA; cheryl.derananian@asu.edu; 2 Food Science & Human Nutrition, Iowa State University, Ames, IA 50010, USA; metisue@iastate.edu; 3 Division of Nutritional Sciences, University of Illinois at Urbana Champaign, Urbana, IL 61801, USA; svthomp2@illinois.edu

**Keywords:** cardiovascular disease, hypertension, stroke, heart disease, diet, health communication, stress, health disparities, blacks, ethnicity

## Abstract

African Americans have a disproportionately higher risk of chronic conditions such as cardiovascular disease (CVD), type 2 diabetes, and hypertension than other ethnic or racial groups. Data regarding CVD-related perceptions and beliefs among African Americans are limited, particularly in the Southwest US. Assessment of current views regarding health and health behaviors is needed to tailor interventions to meet the unique needs of specific populations. We sought to examine knowledge, attitudes, and perceptions of African Americans living in Arizona toward CVD and etiological factors associated with health behaviors and chronic disease development to inform state health agency program development. Transcripts from 14 focus groups (n = 103) were analyzed using Grounded Theory for perceived disease risk, knowledge of CVD risk factors, nutrition, preventative behaviors, and barriers and motivators to behavior change. Participants identified CVD, stroke, and diabetes as leading health concerns among African-Americans but were less certain about the physiological consequences of these diseases. Diet, stress, low physical activity, family history, hypertension, and stroke were described as key CVD risk factors, but overweight and obesity were mentioned rarely. Participants described low socio-economic status and limited access to healthy foods as contributors to disease risk. Focus group members were open to modifying health behaviors if changes incorporated their input and were culturally acceptable. Respondents were 41% male and 59% female with a mean age of 46 years. This study provides insight into CVD and associated disease-related perceptions, knowledge, and attitudes among African Americans in the Southwest and recommendations for interventions to reduce CVD risk.

## 1. Introduction

Cardiovascular disease (CVD) remains the leading cause of death for adults in the United States (US) with an estimated 85.6 million Americans experiencing some form of CVD [1,2]. The term CVD is used to describe disorders of the heart and blood vessels such as coronary heart disease, stroke, congestive heart failure, and arrhythmias. African Americans comprise 13.3% of the US population (46.3 million people) yet have a three-fold greater risk of developing CVD and a two-fold greater risk of CVD-related mortality than that of non-Hispanic whites and other ethnic groups [3,4,5,6]. Coronary heart disease, a condition that originates from atherosclerosis, is the most common form of CVD and the primary cause of one-third of all US adult deaths [7]. This form of CVD remains the leading cause of death for most racial and ethnic groups, including Hispanics, non-Hispanic Whites, and African Americans [5]. Stroke risk among African Americans is 2–3 times higher than that of non-Hispanic Whites and hypertension prevalence is among the highest in the world at 44% [8]. 

The reasons for these disparities in disease risk and mortality are multi-factorial. Differential health care access and quality, environmental or neighborhood influences, persistent racial discrimination, health behaviors such as diet, smoking, socioeconomic status, and genetic variation have been hypothesized as contributors to African American CVD risk [9,10]. Yet, these risk factors are not influential across all situations, and many factors are modifiable, given changed circumstances or behaviors. To lower risk, knowledge and attitudes toward CVD must be assessed to develop intervention programs that will resonate with the target audience [6,11].

Previous CVD risk studies conducted among African Americans have included those living in the US as a whole, or individuals living in states within the American South. These results are limited, as they may inadvertently omit unique characteristics of other regional populations and cultural differences within populations [6,12]. Lifestyle factors in a given region, such as the South, can provide a starting point for new investigations, but may not directly apply to other geographic areas or contexts [13]. Regionally specific qualitative insights into CVD-risk related knowledge among African Americans can fill existing research gaps and permit more accurate targeting of nutrition and lifestyle-related programming [9,14]. For example, dietary interventions based on modification of characteristically Southern or ‘Soul Food’ recipes may not be relevant to all African Americans in or out of the American South where 55% of African Americans reside [15]. Due to differences in geographic origin, life experience, socioeconomic status, and food accessibility, African American culture is diverse in diet and health behaviors [10,13].

In Arizona, African Americans comprise 4.9% of the total population, yet they have the highest CVD-related burden in the state [3,5]. In 2015, the Arizona Department of Health Services reported that the African American CVD risk profile was 41.2% higher than the state average. African Americans lead the state in deaths from heart disease (177.7 cases per 100,000 compared to 145.4 cases per 100,000 for non-Hispanic Whites) [16]. Additionally, African Americans have the highest mortality rate due to stroke (46.7 per 100,000) as compared to other groups in Arizona [16].

One of the first steps to reduce disease burden is to identify sources of health disparities and assess health resources of population groups and communities. However, few studies have evaluated the reasons behind the CVD-related health disparities faced by African Americans in Arizona [9,10]. Qualitative research helps us to understand the unique circumstances that shape and perpetuate poor health outcomes [17,18]. The methodology promotes the expression of opinions and sentiments that may not occur using other approaches like close-ended surveys. Documentation of African American views can aid the biomedical and public health community to better inform, develop, and tailor CVD interventions and programming. The most effective public health interventions are those that resonate with a cultural group and are perceived as needed [10,19].

The purpose of this mixed methods study was to examine the perceptions and knowledge of African American adults regarding heart-healthy behaviors and etiological factors associated with heart disease and other chronic diseases through focus groups (FGs) and self-administered questionnaires. The research objectives were to determine participant knowledge, attitudes, and perceptions of: (1) leading health concerns and causes of death within the African American community; (2) CVD and CVD risk factor etiology; (3) healthcare access concerns among African Americans in Arizona; and (4) the presence of and need for community-based programming for CVD prevention. Study findings were provided to the Arizona Department of Public Health to address health disparities in CVD with African Americans in Arizona. Other research findings on specific food intakes and physical activity among FG participants have been presented elsewhere [20,21].

## 2. Methods and Study Design

### 2.1. Participant Recruitment

Participants were eligible for the study if they self-identified as African American or Black, were between the ages of 25–60 years, and resided in Arizona at the time of recruitment. Potential participants were contacted through email listservs, civic organizations, snowball sampling, and agencies that served the African American community. Flyers were distributed in community locations and in surrounding areas where the FGs were to be held. An online survey service (Survey Monkey) was used to send email invitations and to pre-screen potential participants for eligibility. More than 406 persons contacted the researchers via email for participation. Another 35–40 persons were recruited verbally through snowball sampling or in person at study sites. Screening information was sorted by gender, age group, preferred locations, and days and times to compile groups of 5–10 participants for each FG and a list of alternates. The day before the session, participants were sent a reminder email and contacted by telephone to confirm their attendance, location, and time. If a potential participant cancelled, an attempt was made to find a replacement from the list of alternates.

### 2.2. Focus Group Procedures

Fourteen FGs were conducted with African Americans in Mesa, Phoenix, Tucson, and Casa Grande, Arizona between January and February 2009. Discussions were held at community centers, and public library meeting rooms. Sessions lasted 1–1.5 h. Only the research team and consented participants were allowed in the room. To enhance participant comfort and to keep conversations relevant to their life stage, FGs were divided, in most cases, by gender and age (25–39, or 40–60 years). For two scheduled FGs with men, only one man attended at each site. Data from these two individual interviews were retained for subsequent analysis. All participants provided written, informed consent. A $25 gift card to a major retailer was provided as an incentive at the end of the FG session. This study was approved by the Arizona State University Institutional Review Board (NTS0017).

FGs were led by female African American graduate student moderators who were trained in qualitative methods by the lead author (CD). Moderators had no prior relationships with participants but were familiar with African American informal social networks in Arizona. A standardized moderator’s guide was developed based on a review of the published literature and the researchers’ previous work in African American communities. The moderator’s guide was pilot tested with five African American women aged 22–29 for clarity of questions and reviewed for cultural appropriateness. The research staff continually monitored the moderator guide for content and flow.

Digital audio recordings were transcribed verbatim and verified for accuracy prior to coding and analysis in N-Vivo software (V.11, QSR International, Burlington, MA, USA). Consistent with principles of Grounded Theory, an inductive and iterative approach was used during the coding and analysis process [22]. This analytical approach was selected to ensure identified themes emerged from the data and reflected the views of the participants rather than preconceived notions of the investigators. Two researchers (CD and ST) independently coded each transcript and came to consensus regarding final code allocation. Open coding was initially used to identify the themes and categories within the data, followed by axial coding to confirm that the coding fit the concepts stated in the FGs and to examine how the codes related to one another [22]. Saliency or meaningfulness of the codes was examined by frequency of discussion within and across FGs.

### 2.3. Self-Administered Questionnaires

Participants completed a series of questionnaires upon arrival at the FG venue. Demographic information included age, education, marital status, household income, and self-reported height and weight. Questions from an American Heart Association national survey on women’s health risks (cholesterol knowledge, smoking status, exercise), degree of concern about health conditions, access to healthcare services, and familiarity with heart disease programs targeted towards African Americans were adapted in wording for use with women and men [10,23].

## 3. Results

Demographic information for FG participants (n = 103) is displayed in Table 1. Mean participant age was 46 ± 10 overall (33 ± 4 for 25–39 cohort; 51 ± 6 for 40–60 cohort). Chi-square analysis showed several significant differences by age cohort and gender. Education level and marital status varied by age cohort but did not differ by gender. A larger proportion of the older cohort had some level of college education, whereas more of the younger cohort had completed advanced degrees (*p* = 0.019). A greater percentage of the younger cohort were single (49% vs. 35%), and a larger portion of the older group were divorced, separated, or widowed (6% vs. 28%; *p* = 0.029). Although 11% preferred to not report income, 56% of the respondents earned less than $50,000 per year (n.s.).

### 3.1. Health Risk Factors and Concerns

Table 2 shows the frequency of health risk factors and behaviors by gender and age cohort. BMI, calculated from self-reported height and weight, was significantly greater among women as compared to men (*p* = 0.047). Although 62% of all participants reported they never smoked cigarettes, current smoking rates were higher among men compared to women (45% vs. 4%; *p* < 0.001). A higher percentage of older (63%) vs. younger (31%) participants reported at least one previous discussion about heart disease with a physician (*p* = 0.002). More women than men had received a recent blood test for cholesterol (*p* = 0.018). Most respondents indicated they had health insurance (78%) and that finances had not restricted their ability to seek medical care in the past 12 months (79%).

To quantitatively assess their degree of perceived disease risk and susceptibility, participants were asked how much they worried (a lot, a little, do not worry at all) about 10 diseases or risk factors [9,23]. Nearly 20% of the participants “worried a lot” about heart disease and diabetes, and almost 15% “worried a lot” about stroke and cancer. Perceived worry for breast cancer (*p* < 0.001), HIV/AIDS (*p* = 0.010), lung cancer (*p* = 0.047), smoking (*p* = 0.001), and drug/alcohol addiction (*p* = 0.008) differed by gender. A higher percentage of the younger cohort (23%) than the older cohort (6%; *p* = 0.026) reported they worried a lot about HIV/AIDS. Breast cancer concern differed by age cohort (*p* = 0.049) among women. No other significant differences were seen by gender or age (Table 3).

**Results from the focus group discussions** (Supplemental illustrative quotes are in Supplementary Materials).

### 3.2. Health Concerns

FG participants consistently identified several illnesses as “priority health” issues among African Americans, including heart disease, diabetes, cancer, HIV or AIDS, and hypertension. Participants often mentioned more than one illness when describing priority health concerns. Discussants described the relationship between lifestyle behaviors and genetics or family history in the etiology of these diseases. They also spoke of a general awareness of the connections between disease states, such as hypertension leading to stroke. Expressions of the need for preventive behaviors and early intervention were common.

“*…I think there are a lot of health issues that hit us way more than other communities, not just heart disease, but we’re at a higher risk for HIV. We’re at a higher risk for many different types of cancer…*”

(Male, 25–30, Phoenix)

### 3.3. Leading Causes of Death in African Americans

The diseases or conditions that emerged as leading causes of death among African-Americans were similar to the priority health concerns and conditions described by FG participants: heart disease, hypertension, cancer, diabetes, HIV or AIDS, and stroke. Participants voiced concern that the greater risk of acquiring these diseases was related to health behaviors. Across FGs, it was emphasized that increased disease risk was due to stress and lifestyle factors. Elevated disease risk was attributed to issues related to healthcare access, health education, and availability of healthy foods in their communities. Few participants perceived African American genetics or family history as a reason for the higher disease rates, with the exception of sickle cell anemia. Several participants mentioned HIV and AIDS as a problem that was not openly discussed in the African American community.

“*I think it’s heart disease, prostate cancer, diabetes, and one that I say is silent because we don’t talk about it as much, is HIV and AIDS.*”

(Female, age 40–60, Phoenix)

### 3.4. Healthcare Quality

Participants indicated that access to quality healthcare was a concern. When asked about what influenced the quality of their healthcare, the majority expressed that having high quality health insurance, such as an employer-based group health plan, resulted in good healthcare regardless of skin color. Having more comprehensive health insurance was perceived as advantageous for accessing satisfactory or high-quality health care. For example, it was believed that having comprehensive health insurance affords individuals who are dissatisfied with the care they are receiving from their doctor the opportunity to find another healthcare provider to satisfy any healthcare needs. Without adequate health insurance coverage, which included being uninsured or having insurance through a state provided health insurance plan (e.g., Arizona Health Care Cost Containment System or AHCCCS), the perceived likelihood of receiving quality healthcare was marginal at best. Being underinsured or uninsured was viewed as reducing access to quality healthcare providers and to treatment in general, as illustrated in the quote below. These issues relate to limited income and the general financial restrictions of almost 50% of the FG cohort.

“*I don’t think it has nothing to do with you being Black. I think it has something to do with that insurance card, because they don’t care if you’re purple… They don’t ask you, ‘What color are you?’ They say, ‘Who do you have insurance through?’ And once you say, ‘Nobody,’ you’re in that room a little longer waiting for results.*”

(Male, age 25–39, Phoenix)

FG participants expressed that health insurance played a role in doctor–patient relationships as it influenced the amount of time the doctor spent with the patient and when the patient was able to seek and obtain care. Participants indicated having access to healthcare allowed one to establish a regular, consistent relationship with a physician. In contrast, those who were uninsured or underinsured often had tremendous wait times and were not able to be seen for a condition until it had worsened, negatively affecting the patient-physician relationship. Several younger male participants indicated that they were less likely to seek out routine healthcare and would not go to the doctor unless they were sick.

Although most participants felt access to adequate health insurance was the larger issue, there were some indications that connecting with a doctor they felt comfortable with remained a struggle. This difficulty in finding a doctor was multi-faceted. Several participants stated they tried to find healthcare providers who were persons of color because they believed these healthcare providers would relate to them better. Others expressed that the healthcare received from non-minority doctors was based on stereotypes. For example, one participant discussed how a doctor was insistent that she had high blood pressure because “all African Americans had high blood pressure.” However, other contributors specified that all doctors stereotyped patients to a certain degree. Examples of other stereotypes by doctors were those based on an individual’s weight, age, or income level. Discussants stated finding a minority healthcare provider in Arizona was challenging because the African American population within the state is so small. However, others felt that this was slowly improving as more African Americans were moving to Arizona. Additional selected quotes about healthcare access and insurance are shown in Supplementary Materials.

### 3.5. Knowledge, Attitudes, and Beliefs about CVD

Participants gave a wide variety of responses about the meaning of heart disease. The majority did not provide a medical or mechanistic definition for heart disease (e.g., atherosclerosis, blocked artery due to plaque formation, or inflammatory processes), although participants in 5 of the 14 FGs identified heart disease as a buildup of plaque or a blocked artery. In four FGs, participants openly expressed their lack of knowledge regarding these definitions. Moderator observations suggest that people in several of the other groups were unaware of the definition of these diseases or risk factors and chose to remain silent. One participant communicated a common theme of a biological system unable to tolerate more stress.

“*It’s a buildup of plaque and-and cholesterol. And your heart can only function so much, so there’s more stress on it. And, uh, when your heart is—when the stress is there, uh, you’re just not able to function like you normally would be because of all that stress on your heart.*”

(Female, age 25–39, Phoenix)

Most participants associated “what it means to have heart disease” with lifestyle choices or having other heart related conditions or chronic illnesses (e.g., heart attack, stroke, hypertension, diabetes, high cholesterol). There were frequent references to the role of diet, exercise, not being proactive about one’s health, and lack of education in CVD development.

Few discussants could explain the different types of stroke (e.g., ischemic or hemorrhagic stroke), or what causes a stroke. The FG participants were, however, able to identify stroke symptoms. Commonly mentioned symptoms of a stroke included, “paralysis,” “slurred speech,” “numbness,” and “impaired movement on the left side of the body.” In contrast, other FG participants incorrectly identified chest pain and shortness of breath as symptoms of a stroke. The older FG participants were more aware of stroke symptoms due to personal experiences, such as observing a stroke in a friend or family member. 

Participants were asked to define hypertension, symptoms of hypertension, and diagnostic cutoff points. Only a few participants were able to define hypertension from a medical perspective. In general, there was a lack of understanding among participants about high blood pressure or hypertension suggesting a need for expanded education on this topic.

### 3.6. Perceived CVD Risk Factors

Participants were asked to identify heart disease risk factors and diseases they perceived as related to heart disease development. Participants identified salient modifiable causes of CVD and associated it with several lifestyle choices including poor nutrition, physical inactivity, stress, smoking, and overweight or obesity. Often the lifestyle choices were mentioned in tandem, such as poor diet and physical inactivity. FG discussants frequently mentioned that people had multiple risk factors which contributed to heart disease development. Non-modifiable risk factors that emerged included genetics or heredity and socioeconomic status.

Perhaps the most prominent risk factor across FGs was the role of diet and proper nutrition in the development and/or prevention of heart disease. Diet was mentioned as a CVD risk factor over 100 times across 14 groups. Participants suggested that “not eating right” and “bad dietary habits” were responsible for the development of heart disease, while a high-fat diet was identified as a key contributor. Specifically, participants told us that eating fruits and vegetables, whole grains, foods that are high in dietary fiber, low-fat foods including lean sources of protein, organic foods, and lower sodium foods reduce heart disease risk. There were consistent perceptions about which foods were unhealthy. These included items that were processed, high fat, or fried, red meat or pork, and fast foods. Comprehensive coverage of diet, nutrition and foods is beyond the scope of the current paper and has been discussed in part elsewhere [20,21].

“*A lot of whole grains are very good. It’s probably easier to say what’s not good than what is good but just to try to focus on positive things that are good are going to be fresh vegetables and fruits, less processed food, less fatty foods. Try to avoid anything with white flour, and white vegetables like the corn or the white onions. Just—just think of more colorful things are usually better, if they’re prepared properly.*”

(Female, age 40–60, Casa Grande)

Many participants indicated that they consumed a high fat diet due to traditional African American foods, highly available fast food, and a lack of affordable healthy choices. The traditional or ‘Soul Food’ preparation techniques with added fat or lard (e.g., adding ham hocks or bacon to greens or frying chicken) were perceived to reduce diet quality. FG participants suggested baking, instead of frying, foods as one way to ensure food is healthier. Another recommendation for consumption of healthy foods was substituting traditional recipes with replacements items, such as swapping turkey for ham hocks and olive oil for bacon when cooking green beans. However, some FG participants expressed their concerns about the cultural acceptability of modified recipes, and flavor loss of the original dish if these changes were made, as shown below.

“*…when you cook Soul Food, meals are cooked like for, you know, hours basically—they’re lifeless. So that’s the problem. And then on top of that, people add the butter, the pork, the grease, and all of those things on top of it…*”

(Male, age 40–60, Phoenix)

Other foods recognized as potentially bad for one’s heart included fast foods and processed foods such as those made with high fructose corn syrup, white flour, and sugar. Participants postulated that fast foods and highly processed foods were eaten in the community due to low cost, convenience, and widespread availability. There was also a general perception that healthier foods were more expensive, which was thought to contribute to the inadequate dietary choices made by African Americans. Younger participants were more likely to mention cost as a reason for their dietary choices than the older cohort. Some participants stated that they knew which foods they should be eating, but chose out of convenience, preference, or for economic reasons to eat foods that were not necessarily healthy. These sentiments were more commonly expressed among the younger FG participants. Having a lower socio-economic status was perceived as a CVD risk factor as it was thought to limit healthy food choices and act as a barrier to accessing medical care.

“*And diet’s going to be the big one. Eating poorly is very inexpensive. And that’s going to run prominently in the African-African American community all over the place. They have less funds, so they eat the cheapest stuff they can find, which—it’s not good for you.*”

(Male, 25–39, Phoenix)

Stress was the second most commonly mentioned risk factor for heart disease. Stress was reported to stem from one’s job, living in poverty, experiencing prejudice, having too many commitments, or a combination of factors. Respondents were aware that being African American brought more stress and that difficult economic times aggravated these conditions.

“*There’s the stress of being Black in America that nobody’s writing about, or being a minority in America that, nobody’s writing about—of being a certain minority in America. And, you know, I’m not one to-to blame anybody or play the race card, but in my life experience, it’s just a part of reality.*”

(Female, age 40–60, Tucson)

“*I think, uh, stress. A lot of times we feel like we have a lot of stress going on in our lives and everything. So, when there is… no outlet people tend to hold things in. And that leads to heart disease. To stress. To high blood pressure. Things of that nature.*”

(Male, age 38–60, Tucson)

Physical inactivity was the third most commonly mentioned CVD risk factor. Participants were typically brief in their description of the role of exercise as a risk factor for heart disease, stating that it was a lack of exercise that caused heart disease. There were few references to how exercise reduces CVD risk or improves overall health. As with diet and stress, culture or the social environment and income level emerged as influential factors for making exercise a habitual practice. Concerns about balancing multiple family and work roles due to low income were also mentioned as complicating one’s ability to succeed at achieving adequate physical activity. Respondents across groups suggested that one’s neighborhood location and social environment influence physical activity and that role models and social support facilitate these behaviors.

“*Try to get some physical exercise every day, or most days—at least five or six days a week. It’s sometimes hard because of other commitments, you know, for family and your work and all that at the end of the day to take care of yourself or to get up early to take care of yourself, but some kind of physical exercise.*”

(Female, age 40–60, Phoenix)

“*I think a lot of it is cultural… you’re either in one of … in one of two camps when you’re African American. You’re either working hard, trying to get ahead. You don’t have time to go out jogging for the fun of it. Or you’re not working at all, in which case you don’t care … You’re not motivated to get up and exercise.*”

(Male, age 25–39, Phoenix)

### 3.7. Suggested Strategies to Prevent CVD

FG participants described several health behavior changes to prevent CVD, including physical activity, eating a “good” diet, obtaining regular check-ups and screenings, and stress management. The FG participants often indicated multiple behaviors that needed to be changed and that a lifestyle approach involving changes in multiple health-related domains should be taken to prevent heart disease. Another aspect of prevention pertained to “mindset”. Participants in six FGs called attention to the importance of having a positive attitude toward life and health as a component of preventing heart disease. They also suggested that wanting to change or being motivated to change was necessary for halting CVD-risk enhancing health behaviors. Although less frequently mentioned than the behavioral strategies, participants expressed the need for education about risk factors for heart disease, dietary practices to prevent heart disease, and to a much lesser extent, about which exercises are beneficial for heart disease.

“*I would say attitude, because a lot of times your attitude about something will tell you—will sway you basically if you’re gonna do something about it or not. Are you positive about it or negative about it?*”

(Female, 25–39, Phoenix)

### 3.8. Community-Based Programs and Messaging for Heart Disease Prevention

Participants were asked to describe heart disease prevention programs that were currently available to them, the types of programs they would like to see made available, the types of information they would like to receive, ways to make the information culturally relevant, and what they perceived as the best way to disseminate information on heart disease in an attempt to better understand these factors.

#### 3.8.1. Availability of Programs

FG participants were evenly divided as to whether there were any CVD-related programs available to them. Participants in eight FGs did not know of any programs, and several indicated that they had never seen any programs offered, typically expressing this lack of programs with the simple word, “nothing.” A couple of participants expanded their answer, however, and indicated that part of the problem is that while they are African American, they live in areas that are not predominantly African American.

In contrast, participants in six FGs indicated that there were activities in their community or employer-based health information or programs to prevent heart disease. Participants in four FGs stated that they receive information about health from their church and that churches were doing a lot to improve the health of African Americans. One participant discussed how the Black Nurses Association was partnering with churches to offer programs to African Americans. Other less frequently mentioned community-based resources for heart disease prevention included schools, health fairs, senior centers, and the internet.

#### 3.8.2. Types of Programs Desired and Messaging

In response to the type of programs needed to promote heart disease prevention, a majority of discussants expressed a need for more prevention outreach and awareness and were interested in learning more about CVD themselves. Others indicated that they wanted to learn more about heart disease-related data statistics and to hear from people who have the disease to better understand its consequences. Another suggestion was for education material that not only explained the problem but “offered solutions.”

There was a strong consensus that people would not actively seek out resources to receive information on health and heart disease. There was a sense that programs needed to “reach the people where they are.” Suggested approaches for improving community outreach included offering more church-based health programs, community-based health fairs, and additional health information obtained through flyers, pamphlets, and the internet. Strategies included using different forms of the media to deliver messages, such as ethnically oriented magazines, newspapers, television, the radio and celebrities. Several FGs suggested the internet as a medium to deliver health information and viewed it as easily accessible to most people. Other participants felt that medical professionals, perceived as a “respected” and “trustworthy’ source of information, would be the most appropriate source for health knowledge. Yet, some participants commented that medical professionals are not accessible to everyone. Some of the older women said that it was “too late” to start educating individuals in their age group and that resources should be targeted toward the youth to address potential adverse health behaviors early on so that such changes can make a more substantial benefit. Participants expressed the need for CVD-focused programs to target youth and that these programs can be implemented in schools to influence behaviors from a younger age.

Participants had mixed responses about messages tailored to their race and were split as to whether it was necessary to adapt messages to African Americans. Some felt strongly that health messages did not need to be race-specific. Other participants stated that the messages did not need to be personalized for African Americans, but that there was a need to deliver the information to the African American community. They felt that health information was not necessarily available to everyone who needed it, which was an issue in and of itself.

“*All Americans need information on health care, heart disease, lung, strokes. It’s not a race issue. It’s a people issue.*”

(Male, 40–65, Tucson)

“*…everybody is being affected, you know, and especially diabetes and high blood pressure. All races are being affected.*”

(Male, 25–39, Phoenix)

To make messages more tailored, participants indicated making the information relevant to African Americans and including information that would “catch the eye.” Respondents indicated that not only did current health messages contain images of Caucasian individuals, but they focused on statistics pertaining to whites. It was suggested that the materials had to include both African American images and pertinent statistics. If health messages only contained statistics about other races or images of people from other races, African Americans might believe the illness does not apply to them. In terms of making the materials culturally relevant, it was strongly perceived that it takes more than just including pictures of African Americans to make the materials relevant. Participants indicated that they have different cultural beliefs and engage in different behaviors than other population groups and that these need to be addressed. Some participants felt that a way to connect the message to the community was to deliver it through faith-based organizations. Additionally, some of the participants stressed that the materials needed to be linguistically accurate and not demeaning.

## 4. Discussion

Many African American FG participants had a general awareness of CVD including knowledge about the signs and symptoms of heart attack, stroke, hypertension, and high cholesterol. CVD and related diseases were not the only priority health concerns to emerge from the FGs and surveys, however. Cancer, diabetes, and HIV or AIDS were also viewed as leading causes of death and health worries. These disease rankings were similar to our previous survey results from young adult African Americans in metro Phoenix, and other national surveys with African American women [10,23,24,25].

Despite the participants’ expressed concern about CVD, knowledge of heart disease and related diseases was mixed. Most people could not define heart disease but did correctly associate it with related conditions such as heart attack, stroke, hypertension, high cholesterol, and with unhealthy behaviors such as poor diet, stress, and physical inactivity. While participants were knowledgeable regarding risk factors for heart attacks, stroke, hypertension, and high cholesterol, they expressed a lack of understanding of the pathophysiology and mechanisms behind these conditions. Many did not know the cut-off points for diagnosing hypertension or high cholesterol. They were also limited in their ability to describe the signs and symptoms of heart disease, indicating that for some conditions, they only knew they had these conditions because their doctors told them.

This lack of full understanding can also be confirmed from findings from an earlier national survey inquiring about perceptions and knowledge of heart disease from women, with an over sampling of African Americans. Mosca et al. report only 47% of African American women (n = 130) feel “moderately informed” on heart disease, with 17% “very well informed” and 12% “not at all informed” [24]. A national follow-up survey compared heart disease awareness from 1997 to 2012. These data reflected a continuing gap between Black and non-Hispanic white women. In 1997, 15% of African American women and 36% of non-Hispanic White women were aware that heart disease was the leading cause of death. By 2012, the increase in knowledge rose to 36% for Blacks, but was 65% for Whites [25]. These studies point to reduced heart disease-related knowledge among African American women, which is congruent with the FG results. Clearly more appropriate outreach is needed since African American women remain at higher risk for CVD [6,25].

FG findings indicate a need to provide more education and awareness about what it means when an individual has CVD. This education should include information on CVD signs and symptoms, a basic understanding of CVD risk factors and pathophysiology, and effective prevention strategies. This information needs to be presented in a way that is understandable to the general public, while showing individuals what they can do to prevent and reduce their risk of heart disease using a culturally relevant approach [19,26]. Effective evidence-based interventions should be the starting point for translation and adaptation to cultural settings [27].

Diet, stress, and physical inactivity were identified as salient risk factors for heart disease in the group discussions. Yet, there was a notable disjunction between the FG discussions and the responses on the self-administered questionnaires with many of the participants not practicing healthy lifestyles. This disconnection between knowledge and action underscores the need to enhance opportunities for behavior change. Similar findings were observed with young adult African Americans from the same Arizona communities in a previous study [10]. This dissonance between knowledge and behavior is not unique to African Americans, Arizonans, or by socioeconomic status. In a New York blood pressure intervention trial, African Americans did not meet recommendations for physical activity or fruit and vegetable intake, despite high health behavior knowledge [28]. Predominately white, higher income, college-educated women in Arizona misinterpreted what foods comprised a low carbohydrate diet even with information access and economic resources [29]. Multiple studies have highlighted the gaps between intrinsic motivation, knowledge, and health behaviors [30].

Qualitative inquiry can be the best approach for identifying the unique barriers to and motivators for sustainable change [26,31]. As such the detail provided by respondents on preferred program types can be useful to generate possibilities for CVD education. Drawing on African American family and cultural practices through hands-on cooking and group workshops could be a successful way to connect people with their culture, food, and health [32]. Sharing stories and building community through food ways is a part of African American culture that would likely be well received based on participant views.

It is notable that overweight and obesity were rarely mentioned in FG discussions as a factor in CVD or health risk. Other research has suggested that African Americans may be more comfortable with their bodies regardless of medical or mainstream social norms regarding body size [33]. Considering that 64% of the participants were overweight or obese, it may be that depictions of thinner non-African Americans may be viewed as a cultural ‘other’ and not desired or obtainable [33,34].

Stress produced by one’s job, poverty, having too many commitments, experiencing prejudice, or a combination of factors was recognized as associated with the development of CVD. Many participants indicated that African Americans led particularly stressful lives. These discussions frequently centered on economic limitations, rather than racial discrimination. It is important to note the complex interactions between socioeconomic status, race, gender, and age in CVD risk [30,32,35]. Healthcare access was expressed as an urgent African American health issue. Many participants indicated that they lacked adequate health insurance coverage which, in turn, influenced the type of healthcare they were able to receive, and added to stress.

Non-modifiable risk factors for heart disease, such as socio-economic status and heredity, were also discussed. Those with a lower socioeconomic status were perceived as having more life stressors and reduced access to health insurance and healthcare, healthy food choices, and places to be physically active. Participants identified lower socio-economic status as a potential precursor to not only heart disease but to engaging in fewer healthy behaviors. This provides implications in terms of possible interventions. While socio-economic status is not always modifiable, interventions can focus on ways to improve dietary quality, how to locate healthy, affordable foods, and how to engage in low-cost physical activities. Interventions can also focus on enhancing healthcare access among low income individuals through screenings and improving connectivity to healthcare. Pace et al., found that rural African Americans in Alabama felt that lack of income directed one’s focus on daily activities rather than preventative health practices [36].

Genetics or heredity was considered a significant risk factor in CVD, but it was also suggested that genetics could be overcome by engaging in healthy behaviors. It was expressed that behaviors are passed on within families and that this may drive the apparent association between genetics/heredity and heart disease.

Strengths of the current study include the large sample size for qualitative analysis, breadth of perspectives from both men and women, young adult through middle age, and variety of geographic locations in Arizona. There are several limitations to be noted. Perceptions and experiences of the participants may be different from those who did not engage in the research study. The nature of FG methodology is such that quotes are not linked to the quantitative survey responses of individuals. Our qualitative and quantitative results may not represent all African American’s views in Arizona or nationally.

## 5. Conclusions

Consistent with national data on cardiovascular disease and other chronic illnesses, FG participants recognized African Americans were at higher risk for numerous chronic illnesses including heart disease, stroke, cancer, diabetes and HIV/AIDS. Participants clearly recognized the need to intervene on lifestyle factors related to the development of these chronic conditions. Responses regarding the etiology and consequences of cardiovascular diseases, as well as the lifestyle behaviors associated with increased cardiovascular risk, suggest the need for increasing the availability of educational programming. Medical professionals were perceived as the most appropriate source of health information despite discussion on issues with adequate healthcare.

Suggested avenues for heart disease prevention programs in the African American Community included outreach and awareness programs, health fairs, and programs offered through schools and churches. Relevant topics include information regarding strategies for heart disease prevention, the signs and symptoms of heart disease, and feasible approaches for making behavior changes. It should also be noted that an important viewpoint also emerged: heart disease is a problem that touches all humans regardless of race or ethnicity and making the information available is most important.

While participants were mixed in their opinions about whether the materials and programs needed to be specifically tailored to African Americans, key strategies were identified for tailoring the intervention materials, including disseminating the information through ethnically relevant images, statistics, media, health behaviors, health concerns, and language. It was suggested to engage trusted members of the African American community in the delivery of health messages, especially when suggesting health behavior changes and recipe alterations.

The findings from this study can be used to help create targeted interventions, particularly with low income African Americans. One important avenue to address CVD risk is through culturally appropriate nutrition and shared experiences [31]. Program development could build upon existing knowledge of unhealthy versus healthy dietary habits and provide solutions for dietary change. Reducing stress and increasing physical activity are also important avenues for intervention. An upstream approach, of influencing environmental, social and socio-economic determinants of health as well as individual level approaches focused on education and behavior change may be most effective in addressing the complex influences on CVD risk factors and health behaviors identified in this study.

## Figures and Tables

**Table 1:** Distribution of demographic characteristics of Arizona African American focus group participants by gender and age group [*n* = 103; *m* ± SD; % (*n*)].

	Total (103)	Male 41% (42)	Female 59% (61)	25–39 Years 34% (35)	40–60 Years 66% (68)
Age in years (*m* ± SD)	46 ± 10	43 ± 10	46 ± 10	33 ± 4	51 ± 6
**Education**					
No High School/GED	8% (8)	10% (4)	7% (4)	14% (5)	4% (3)
High School Grad/GED	5% (5)	10% (4)	2% (1)	6% (2)	4% (3)
Some College—no degree	31% (32)	38% (16)	26% (16)	^†^ 14% (5)	^†^ 40% (27)
Associates	10% (10)	4% (4)	10% (6)	9% (3)	10% (7)
Bachelors	24% (25)	12% (5)	33% (20)	20% (7)	27% (18)
Masters/Doctoral/Professional	22% (23)	21% (9)	23% (14)	^†^ 37% (13)	^†^ 15% (10)
**Marital Status**					
Single	40% (41)	48% (20)	34% (21)	49% (17)	35% (24)
Married/Live with partner	40% (41)	31% (13)	46% (28)	46% (16)	37% (25)
Divorced/Separated/Widowed	20% (21)	21% (9)	20% (12)	^†^ 6% (2)	^†^ 28% (19)
**Household Income**					
<$10,000–14,999	19% (20)	29% (12)	13% (8)	11% (4)	24% (16)
$15,000–29,999	13% (13)	12% (15)	13% (8)	9% (3)	15% (10)
$30,000–49,999	24% (25)	19% (8)	28% (17)	23% (8)	25% (17)
$50,000–74,999	12% (12)	7% (3)	15% (9)	11% (4)	12% (8)
$75,000–99,999	13% (13)	14% (6)	11% (7)	9% (3)	15% (10)
$100,000 +	9% (9)	5% (2)	12% (7)	17% (6)	4% (3)
Prefer not to answer	11% (11)	14% (6)	8% (5)	20% (7)	6% (4)

Significant differences are indicated by ^†^ *p* < 0.05 for age cohort.

**Table 2:** Health behavior characteristics of focus group participants [*n* = 103; *m* ± SD; % (*n*)].

d	Total (103)	Male 41% (42)	Female 59% (61)	25–39 Years 34% (35)	40–60 Years 66% (68)
**BMI Category (%)**					
Normal 18.5–24.9	36% (36)	50% (21)	26% (15)	44% (15)	32% (21)
Overweight 25.0–29.9	28% (28)	24% (10)	31% (18)	18% (6)	32% (21)
Obese 30.0–34.9	22% (22)	32% (7)	26% (15)	26% (9)	20% (13)
Class II+ obesity ≥ 35.0+	14% (14)	10% (4)	17% (10)	12% (4)	15% (10)
**Exercise frequency**					
Almost never	14% (14)	7% (3)	18% (11)	14% (5)	13% (9)
Twice a month	20% (21)	21% (9)	20% (12)	20% (7)	21% (14)
Once a week	11% (11)	10% (4)	11% (7)	17% (6)	7% (5)
2–3 times per week	21% (22)	17% (7)	25% (15)	26% (9)	19% (13)
4 or more times per week	34% (35)	45% (19)	26% (16)	23% (8)	40% (27)
**Smoking Frequency** *					
Never smoked	62% (64)	45% (19)	74% (45)	60% (21)	63% (43)
Smoke, but successfully quit	17% (18)	10% (4)	23% (14)	11% (4)	21% (14)
Smoke, <10 cigarettes per day	10% (10)	21% (9)	2% (1)	14% (5)	7% (5)
Smoke, >10 cigarettes per day	11% (11)	24% (10)	2% (1)	14% (5)	9% (6)
**Dr. has discussed heart disease and health w/you ^†^**					
Yes	52% (54)	57% (24)	49% (30)	31% (11)	63% (43)
No	48% (49)	43% (18)	51% (31)	69% (24)	37% (25)
**Cholesterol check in the past 18 months** *					
Yes	70% (70)	56% (22)	79% (48)	58% (19)	76% (51)
No	30% (30)	44% (17)	21% (13)	42% (14)	24% (16)
**Blood pressure check in the past 18 months**					
Yes	88% (91)	86% (36)	90% (55)	80% (28)	93% (63)
No	12% (12)	14% (6)	10% (6)	20% (7)	7% (5)
**Healthcare coverage (general)**					
Yes	78% (80)	74% (31)	80% (49)	89% (31)	72% (49)
No	22% (23)	26% (11)	20% (12)	11% (4)	28% (19)
**One person as personal Dr. or healthcare provider ^†^**					
Yes	74% (64)	56% (20)	86% (44)	60% (18)	81% (46)
No	26% (23)	44% (16)	14% (7)	40% (12)	19% (11)
**Past 12 months needed to see doctor, but did not have money**					
Yes	21% (18)	14% (5)	26% (13)	20% (6)	21% (12)
No	79% (69)	86% (31)	74% (38)	80% (24)	79% (45)
**How long since last doctor visit for routine checkup**					
Less than 12 months ago	74% (64)	61% (22)	82% (42)	60% (18)	81% (46)
1 year but < than 2 years ago	12% (11)	17% (6)	10% (5)	20% (6)	9% (5)
>2 years ago	14% (12)	22% (8)	8% (4)	20% (6)	10% (6)

Significant differences are indicated by * *p* < 0.05 for gender, and ^†^ *p* < 0.05 for age cohort.

**Table 3:** Degree of worry about developing a health condition among African American focus group participants by gender and age cohort [*n* = 103; % (*n*)].

Health Conditions	Total (103)	Male 41% (42)	Female 59% (61)	25–39 Years 34% (35)	40–60 Years 66% (68)
**Heart disease/heart attack**					
Worry a lot	19% (19)	22% (9)	16% (10)	20% (7)	18% (12)
Worry a little	48% (49)	49% (20)	48% (29)	46% (16)	49% (33)
Do not worry at all	33% (34)	29% (12)	36% (22)	35% (12)	33% (22)
**Diabetes**					
Worry a lot	18% (18)	17% (7)	18% (11)	23% (8)	15% (10)
Worry a little	48% (50)	45% (19)	51% (31)	54% (19)	46% (31)
Do not worry at all	35% (34)	38% (16)	31% (19)	23% (8)	40% (27)
**Stroke**					
Worry a lot	15% (15)	19% (8)	12% (7)	20% (7)	12 % (8)
Worry a little	42% (43)	43% (18)	41% (25)	40% (14)	43% (29)
Do not worry at all	44% (45)	38% (16)	47% (29)	40% (14)	47% (31)
**Cancer (general)**					
Worry a lot	14% (14)	14% (6)	13% (8)	14% (5)	13% (9)
Worry a little	48% (50)	52% (22)	46% (28)	57% (20)	44% (30)
Do not worry at all	44% (45)	33% (14)	41% (25)	29% (10)	43% (29)
**HIV/AIDS** *,^†^					
Worry a lot	12% (12)	12% (5)	11% (7)	23% (8)	6% (4)
Worry a little	17% (18)	31% (13)	8% (5)	20% (7)	16% (11)
Do not worry at all	71% (73)	57% (24)	80% (49)	57% (20)	78% (53)
**Smoking** *					
Worry a lot	10% (10)	17% (7)	5% (3)	14% (5)	8% (5)
Worry a little	13% (13)	24% (10)	5% (3)	11% (4)	14% (9)
Do not worry at all	77% (78)	59% (24)	90% (54)	74% 26)	79% (52)
**Alzheimer’s**					
Worry a lot	9% (9)	10% (4)	8% (5)	14% (5)	6% (4)
Worry a little	35% (36)	39% (16)	33% (20)	34% (12)	36% (24)
Do not worry at all	56% (57)	51% (21)	59% (61)	51% (18)	58% (39)
**Breast Cancer** *,^†^					
Worry a lot	8% (8)	0%	13% (8)	0%	12% (8)
Worry a little	38% (38)	18% (7)	51% (31)	49% (17)	32% (21)
Do not worry at all	54% (55)	82% (33)	36% (22)	51% (18)	56% (37)
**Lung Cancer** *					
Worry a lot	7% (7)	12% (5)	3% (2)	9% (3)	6% (4)
Worry a little	25% (26)	33% (14)	20% (12)	31% (11)	22% (15)
Do not worry at all	68% (69)	55% (23)	78% (46)	60% (21)	72% (48)
**Drug addiction or alcoholism** *					
Worry a lot	5% (5)	5% (2)	5% (3)	6% (2)	4% (3)
Worry a little	14% (4)	26% (11)	5% (3)	14% (5)	13% (9)
Do not worry at all	82% (84)	69% (29)	90% (28)	80% (28)	82% (56)

Significant differences are indicated by * *p* < 0.05 for gender, and ^†^ *p* < 0.05 for age cohort.

## Supplementary Materials

The following are available online at http://www.mdpi.com/1660-4601/15/11/2433/s1.
