# Supplementary material for: Perceptions of Heart-Healthy Behaviors among African American Adults: A Mixed Methods Study
Source: Int J Environ Res Public Health. 2018 Nov 1;15(11):2433. doi: 10.3390/ijerph15112433 (PMC6265893; doi:10.3390/ijerph15112433)
Supplement: Supplementary file 1 [file ijerph-15-02433-s001.pdf]

Table S1. Selected illustrative quotes from African American focus group participants

Thematic construct	Illustrative quote with gender and age cohort
1. & 2. Perceptions about health concerns and leading causes of death<br>• HIV and AIDS<br>• Type 2 Diabetes<br>• Cancer	"I guess I would have to say it is probably something related to all the things we said. Probably right now, I would think AIDS or possibly hypertension or heart disease, one of those two – three." (Female, age 25-39, Phoenix)<br><br>"I think, uh, sugar diabetes is one -- another one. And my mother has the high blood pressure, you know, and it runs in the family. That's one -- I think that's the number one killer." (Male, age 25-39, Phoenix)<br><br>"I think that a lot of times the-the myth is, is that for Black people -- because I think we have a l-- a lack of health, uh, knowledge -- is that we figure if my mama has this, then I am prone to have it. And I think that's a myth. [If you think], you know, if you continue with the same lifestyle and continue to do the same thing they did, of course you're going to inherit it. But I don't think it's just inherited because you're related to that person." (Male, age 40-60, Tucson)<br><br>"Not taking care of yourself once again. Not being checked out. Because something -- in most cases you can prevent a stroke if you catch it in time. Like my mother. She had two of them. And the first one they didn't give her the proper treatment. The second one they caught it a little late. But they said she had a blood clot in her neck or something and it shut off one side of the brain. So she was paralyzed. And now she only has like 80 percent use of that side -- of the left side of her body" (Male, age 40-60, Tucson)
3. Healthcare quality and insurance	"I think most of us probably think about cancer because cancer can strike at anytime. Often times we have some kind of warning signs. For a lot of people in particular maybe for African Americans maybe due to lack of insurance, they probably don't let their healthcare professional … know about it, they maybe even scared to even go to the doctor to find out what might be wrong." (Male, age 40-60, Phoenix)<br><br>"I think we're also seeing more colon cancer among us women for the same reason -- the lack of screening. Maybe we delay going to the doctor, and more recently with the economy and all, um, the lack of, even if we are employed, the lack of medical insurance and coverage is -- or if we have coverage -- they don't cover certain things. So again it's cost and lack of education or prescreening." (Female, age 40-60, Phoenix)<br><br>"I think, from another perspective, uh, economics, how much money you have available to be able to go out and eat healthy food choices, uh, to be able to go and visit a physician so that you're getting regular checkups to see if, you know, you are starting to exhibit some of these symptoms. And you may just interpret them as something else." (Female, age 25-39, Phoenix)

	"… sometime, you know, we can't see our doctor. Don't have no medical, it's kinda hard." (Female, age 40-60, Casa Grande)<br><br>"I don't think it's because you're Black, I think it's because of insurance when they, you know, the provider cares about the money, really, so you have to wait until you have to go to the hospital where they can't refuse you and you know, causes delays in care which makes problems go on too long before you can do anything about it." (Female, age 40-60, Casa Grande)<br><br>"But then, there's the barriers of, if you are on Medicaid or one of the AHCCCS plans, there's stereotypes with that and -- even from the physician point of view. I know, with me and my private insurance, I get a reminder call." (Female, age 25-39, Phoenix)<br><br>"I think it's the healthcare coverage because I've been fortunate enough to always have good healthcare coverage but a lot of people in my family haven't so we've been to the same doctor and had two totally different experiences, until it was identified oh that's your sister, oh that's your cousin then it's a whole different ballgame" (Female, age 25-39, Phoenix)
4. Beliefs and perceptions about CVD<br>• Causes<br>• Concern with lack of knowledge<br>• Risk factors for CVD<br>o Stress<br>o Health Behaviors<br>o Lack of Knowledge<br>o Heredity	"I was always thinking, I don't know, atherosclerosis always is associated with, cardiovascular disease and obesity is the tie, the blockage of your arteries."(Female, age 25-39, Phoenix)<br><br>"Diabetes, high blood pressure, high cholesterol are related to heart disease." (Female, age 40-60, Casa Grande)<br><br>"I'm going to say high blood pressure. And diabetes too, because they work hand in hand pretty much would be the cause of heart disease and stroke." (Female, age 40-60, Phoenix)<br><br>"…When I think of heart disease I think that it encompasses a whole bunch of other…related illnesses such as strokes, heart attacks, diabetes and all those things contribute to heart disease." (Female, 25-39, Phoenix)<br><br>"It can mean a lot of things. Sometimes it just means something minor or just, you know, they just have a clog. And then they can go up there and, you know, and unclog it. And then sometimes they need a bypass. So I think its extremes and minor things…" (Female, age 40-60, Phoenix)

	"I really don't know what heart disease is but I know that … strokes and heart attacks are related to it." (Female 25-39, Phoenix)<br><br>"Or it could [mean] -- that's what a stroke actually is, that somehow or another, the-the brain had a lack of oxygen and nourishment. It killed that area of the brain. It ruptured the vein or artery in there. (Male, 25-39, Phoenix)<br><br>"My high blood pressure would mean that in some kind of way your heart is pumping blood up and it's not coming down properly. And then it's building up in …the higher area. So that would … probably be too much fluid in the lower extremities you know keeping the blood high where it won't fall -- where it doesn't fall. I don't know." (Male, age 40-60, Phoenix)<br><br>"It means that your heart is having to work a little bit harder to pump that blood through your body, I know you don't want a clinical explanation, but… And so when the heart has to work a little bit harder to pump that blood through your body the contributing factor could be your weight, your stress level that's a precursor to other stuff to start to form that's when you get high blood pressure." (Female, age 25-39, Phoenix)
5. Perceived CVD risk factors<br><br>Nutrition<br>• Strategies for dietary improvement<br>• Barriers<br>• Motivators	"They didn't eat right, or they're not being healthy by staying active. Um, they even eat the wrong type of foods. They're not really staying active or controlling their stress. They get to a point where it's just gotten so bad that -- we wait too late. It's too late, you know, way too late by the time we try to do something about it. (Male, age 25-39, Phoenix)<br><br>"I don't understand about heart disease either. I think, you know, it's our eating habits that causes us to have a bad heart in the first place. Our eating habits and not exercising enough, so I'm really not sure about the – the heart disease, I'm not sure about that." (Female, age 40-60, Casa Grande)<br><br>"I agree, um back again with the high blood pressure, no exercise, eating the wrong foods, no I won't say wrong foods. I'll say red foods, fried foods." (Female, age 40-60, Casa Grande)<br><br>"I think it's our diet, I think it's kind of probably some of the ways that we view things that probably causes more stress than other, that may cause it as well, lifestyle habits, lack of exercise, just not eating right, our heart has to work a lot harder." (Female, age 25-39, Phoenix)<br><br>"I think we're more susceptible because we eat the good stuff, the fried stuff, every day. That's what we can afford." (Male, age 25-39, Phoenix)

	"You know, you grab something, and it, more than likely, is not the appropriate or healthiest choice when you're on the go. But it will suffice. But then, it -- you pay a price down the line." (Female, age 25-39, Phoenix)<br><br>"I think two of the biggest are, um, stress and bad diet. And I know that I'm one of those people that -- I eat a lot of garbage. I eat a lot of junk food. And I'm just not trying to change it right now. I like the stuff I eat." (Male, age 25-39, Phoenix)<br><br>"…I have changed my eating habits in the past. …since I had my heart attack, so I had my bypass, I had to change my eating habits. I had to add more fruits and vegetables to my diet, cut out some starches." (Female, age 40-60, Tucson)<br><br>"Yeah, um… well… when I think of foods that are relatively heart healthy, [I think of] vegetables, and um you know fruits." (Male, age 25-60, Phoenix)<br><br>"And the way it's prepared makes it even more bad because it's either fried, I mean really fried, deep fried or just put out on the grill where it's so greasy and the butter and stuff that we use to cook it with. How its prepared makes it even -- I guess that's why I eat it because it keeps the taste." (Male, age 25-60, Phoenix)<br><br>"I also think that in some communities the lack of available fresh food, vegetables, fruits, and at a reasonable cost when it is available, and income -- it's a lot cheaper, to eat I think, for some families to spend $5 and buy some French fries for $1 and a hamburger -- you know, feed several people -- then it is to buy an orange. That an orange can sometimes cost $1.30 -- just for one orange. So I think income and -- and availability of -- of good, wholesome, fresh foods -- foods -- is an issue." (Female, age 40-60, Phoenix)
Stress	"I think …these issues come up more because of the stressors [we have] as African Americans so I think …statistically speaking our numbers may be a little bit more skewed because we have …other stressors that relate to our health status in this country." (Female, 25-39, Phoenix)<br><br>"… you can actually stress yourself to death because it can cause damage to your arteries. And, you know, it will lead to heart disease or whatever problem. So just anything throughout your day, just normal daily activity you're going to be -- your body's going to be under a certain amount of stress. It goes back to nutrition and-and-and giving your body the nutrients, the antioxidants to combat, you know, the stress-related conditions internally." (Male, age 40-60, Phoenix)

Physical activity<br>• Type/Frequency<br>• Barriers<br>• Motivators	"Growing up, we didn't really have a choice to sit around and watch TV. You had to get out and it was a privilege to go out and run around. You could run -- I mean, ride your bike, run around, go to the center, whatever. And I don't think that children have that freedom today, because we're scared that they're gonna get picked up or whatever." (Female, age 40-60, Tucson)<br><br>"And time. It's hard to get in there with that. 45 minutes doesn't sound like a long time but out of your day, you know, that's probably 45 minutes you could prepare dinner for your kids, or doing homework, or driving home or to work or – time is limited." (Female, age 40-60, Casa Grande)<br><br>"So when you're younger, you're kind of conditioned to be outdoors and playing and having fun. And as you grow older, it's a habit. So you stick to the habit. You're kind of used to it. So now I play ball, not 'cause I'm trying to stay in shape or anything like that, but cause I like playing ball, 'cause I grew up playing ball. So I think a lot of it is opportunity. I mean, it's true. People are busier now than they ever been, you know, working, struggling, you know. But I think if it's part of your routine and part of your habit, it's easier to continue to do it even as your time becomes more constrained." (Male, age 25-39, Phoenix)<br><br>"I think that I think there's a lack of inventiveness or being creative. I know sometimes when I exercise, I just dance for an hour. And, you know, in that process -- it's not like you have to go outside your house and you gotta go to the gym. I mean, you don't have to -- you don't have to do that. And so there's a way to look at it outside of the box of 'I gotta go do this on a treadmill,' which may be discouraging." (Female 40-60, Phoenix)<br><br>"I would say, try to get some physical exercise every day, or most days -- at least five or six days a week. It's sometimes hard because of other commitments, for family and your work and all that at the end of the day to take care of yourself or to get up early to take care of yourself, but some kind of physical exercise." (Female, age 40-60, Phoenix)<br><br>"Time is just a huge factor, because so many people now are hustling just to make it that you're working two jobs, or you're working, and then you get home [and] you have to take care of family. And exercise is just not -- nowhere on your priority list. So, we just have lifestyles and cultures that -- where that is not an important factor. (Male, age 25-39, Phoenix)
6. Strategies to prevent CVD	"It's just that, if you're on a low, fixed income, you are never going to eat healthy unless you're cooking your own food." (Male, age 25-39, Phoenix)

	"Try to cut out certain foods as much as possible, like saturated fats. Try to incorporate some good things and try to find some good guidelines for eating well and follow them as best you can." (Male, 40-60, Phoenix)<br><br>"I think another barrier is self-inflicted. You know, there's that motivation factor because sometimes, once you get home, you don't feel like doing anything. And then also, on the other side of motivation is having a network of people because, if you have other people, you say, 'Okay, we're going to schedule this time.' And it's important for us because prevention on the front end is going to be less expensive than, you know, taking care of something on the backend." (Female, age 25-39, Phoenix)
7. Community based programs and messaging<br>• Lack of other African Americans<br>• Importance of church	"And I know that, like, the Black Nurses Association and some places like that have teamed up with some of the churches, like-like the bigger ones, like, uh, Pilgrims Rest [FIBC, Tanner Chapel]. And they make -- sort of make the rounds, you know, every couple of months…. And they'll do blood pressures. And they'll do some educational programs. I know that. And some of the churches, if you go into the ladies' room, there's all kinds of little pamphlets and stuff about, you know, heart disease, you know, the-the things that affect us most often. AIDS, HIV, heart disease, high blood pressure, those kinds of things." (Female, 25-39, Phoenix)<br><br>"My church does annually do a program. They -- I don't know whether they take the whole month, they do the heart month and do the whole red thing and they spend time talking to the membership about their heart health. And it's a predominantly African American church." (Female, age 25-39, Phoenix)<br><br>"Our church is doing a health/blood drive next Saturday on the 24th and it's gonna be donating the blood and then there will be health booths set up, so yes that is correct, yeah." (Female, age 25-39, Phoenix)<br><br>"My job did a [coronary] study and it actually took into account your race and your age, and then they -- it was a program that you could type things into and they actually worked with people. They didn't have anybody for African-Americans, per se, and they recognized that and at least were open about it, you know, "We don't have anybody that has a specialty in your race, but, uh, we can tell you these things that we do know." And so I thought that was useful." (Female, age 25-39, Phoenix)<br><br>"I think that it should be targeted definitely. There's not a business in this country that does not practice target [marketing]. I mean that is fundamental. I mean everybody -- everybody is targeting a specific group for a specific product. So you don't target this because we're being racist. You're targeting because there are certain different needs in particular groups that are different from others. So definitely it must be targeted." (Male, age 40-60, Phoenix)

	"The media -- some men are not gonna go out and just stick in their own little comfort zone. And maybe, uh, some of the celebrities that have changed their habits -- we all know about Oprah, but I'm sure there's some men, like Montel Williams, he's changed his habits. If they can give spots on ESPN where the men are watching all these sports, that may be a benefit. I know my husband is sticking to his TV and his comfort zone and that's where you can catch him." (Female, age 40-60, Phoenix)<br><br>"I believe you also -- you need to target. If you want to sell any product, you're gonna have to target the market. You can't just say, 'I'm gonna advertise to everybody,' 'cause we turn deaf ears. You have to speak our language to us if you want us to hear."(Female, age 25-39, Phoenix)<br><br>"I do think they should be targeted toward African-Americans. I think that some of the African Americans, it would appeal to them if they were being spoken to by African Americans or about their specific needs. I think that we may have different issues, and there needs to be some sort of education that because of your race and your culture, you're more susceptible to this and that and there are some things that you should be doing." (Female, age 25-39, Phoenix)<br><br>"I would like for it to be put to the forefront of my mind this is more prominent among African Americans, or that we're the most stricken ethnicity to this ailment, you know, it's going to make it seem more important to me. Yeah everybody's getting heart disease but it's more prominent among, you know, this community or another community, same thing. Um, I – it's not the only thing but I do think it makes it more, um, personal for us." (Female, age 40-60, Casa Grande)<br><br>"…like when you watch commercials. I feel that a lot of commercials are racially motivated because of the individuals that they portray in those commercials. So you talking about, say for example diabetes, you know, I know every race gets it but Africans Americans the highest. So why is all the commercials showing Caucasians? So my point is let's gear them a little more, if you put a Black person on there, most African Americans tend to focus a little bit better you know." (Male, 25-39, Casa Grande)
